# A Spatial Analytic Approach to Maternal Health Following Hurricane Florence (2018)

**DOI:** 10.1007/s10995-026-04257-0

**Published:** 2026-04-29

**Authors:** Kristen Lysne, Margaret Sugg, Charlie Reed, Jennifer Runkle, Dennis Guignet, L. Baker Perry

**Affiliations:** 1 Department of Geography and Planning, Appalachian State University, Boone, NC USA; 2 North Carolina Institute for Climate Studies, North Carolina State University, Asheville, NC USA; 3 Department of Economics, Appalachian State University, Boone, NC USA

**Keywords:** SaTScan, Maternal health, North Carolina, Tropical cyclones, Severe maternal morbidity, Hurricane florence

## Abstract

**Background:**

The United States leads developed nations in maternal morbidity, yet research on the literature surrounding severe maternal health in the context of natural disasters remains limited. Projections suggest that tropical cyclone (e.g., hurricane, typhoon, cyclone) intensity will continue to surge as global temperatures rise, and experts warn that they pose one of the most significant threats to global public health in the 21st century.

**Objective:**

This study is the first to apply a spatial clustering approach to maternal health following exposure to a tropical cyclone in North Carolina.

**Methods:**

We conducted an exploratory clustering analysis of hospitalizations for Severe Maternal Morbidity (SMM-21) using the Bernoulli-Kulldorff SaTScan statistic in the context of Hurricane Florence (2018). Multivariate logistic regression identified individual and contextual factors associated with high-risk clusters in the aftermath of Hurricane Florence (2018).

**Results:**

All 28 FEMA disaster-declared counties had presence within an SMM spatial cluster, while individual factors (age ≥ 40) and contextual factors (racial segregation [ICE Race], reduced greenspace, and high-urbanity) were associated with residence in high-risk clusters.

**Conclusion:**

Results indicate the importance of a spatial analytic approach following climate disasters to better identify characteristics of high-burden maternal populations for post-disaster relief and response.

**Supplementary Information:**

The online version contains supplementary material available at 10.1007/s10995-026-04257-0.

## Significance

The aim of this study is to explore the association between hurricane exposure and the adverse maternal health outcome severe maternal morbidity (SMM). We used a Bernoulli distribution, SaTScan spatial analysis, and multivariate regression techniques for SMM to assess trends after hurricane exposure. We use regression analysis and SaTScan spatial analysis to identify the individual and contextual drivers of high-risk residence. Residence in a FEMA-declared disaster county, residence in racially segregated and age over 40 were significant drivers of high-risk SMM clustering.

**Supplementary Information:**

The online version contains supplementary material available at 10.1007/s10995-026-04257-0.

## Introduction

Between 2004 and 2013, land-falling hurricanes (tropical cyclones with maximum sustained winds > 119 km/hr) are a leading cause of natural disaster related fatalities in the United States and, compared to other natural disasters, resulted in the highest economic losses, totaling $392 billion. Beyond immediate fatalities, the consequences include enduring health risks from property damage, infrastructure loss, economic disruption, and environmental degradation (USGCRP 2016). Tropical cyclone (TC) activity in the past 50 years has been above the 20th-century average, with approximately eight TC events per 5-year period (Kunkel and Easterling 2020). Projections suggest that TC intensity and rainfall will continue to surge as global temperatures rise, with experts warning that TCs pose one of the most substantial threats to global public health in the 21st century (Atwoli et al., 2021).

Separately, the United States has also experienced a significant increase in its maternal mortality rate, from 17.4 maternal deaths per 100,000 live births in 2018 to 23.8 deaths in 2020 (Sonenberg, 2023), ranking the U.S. as the highest among developed nations. Pregnant and postpartum individuals are highly susceptible to the effects of natural disasters and extreme events (Silva-Suarez et al., 2021), and the Environmental Protection Agency (EPA) identifies them as a high-risk demographic group for health impacts from extreme weather events (EPA, 2017). Climate change is also an emerging threat to maternal health (Veenema et al., 2023; Sbiroli et al., 2022; Barkin et al., 2022). Together, these trends demonstrate the need for prevention and response efforts centered on maternal health in the context of climate-driven disasters. 

Severe maternal morbidity (SMM) referes to unexpected outcomes of labor and delivery that result in short- or long-term consequences for an individual’s health (CDC 2023), and SMM are increasing in the United States. Underlying health conditions as well as environmental and social factors have been linked to higher SMM risk (Harden et al., 2022). Previous research has examined the impacts of heat (Jiao, Sun, Avila, 2023; Harden et al., 2022; Ulrich et al., 2026) and flooding (Sugg et al. 2023a, 2023b) on maternal health indicators such as SMM, studies on the influence of TCs on SMM outcomes remain notably absent from the literature. This study uses spatial analysis to identify TC-impacted areas and evaluates geographic patterns in SMM and TC exposure to investigate the underlying social determinants that increase risk. Results highlight geographic patterns of SMM following tropical cyclone exposure and identify community- and individual-level determinants associated with high-risk areas, informing targeted disaster preparedness for maternal populations.

## Research Objective and Scope

There is a need for geographically informed analyses of maternal health outcomes following catastrophic environmental events, as no studies have yet examined spatial patterns in post-TC maternal health. This exploratory study applies a spatial epidemiological approach, using Kulldorff’s spatial scan statistic within a reproductive justice framework, to assess the spatial patterns of maternal health and how these patterns changed before and after Hurricane Florence (2018). More specifically, it aims to examine the spatial patterns of SMM after Hurricane Florence (2018) and assess underlying community and individual determinants influencing these patterns. Results will inform which maternal populations are most at risk during extreme weather events like Hurricane Florence.

## Methodology

### Study Population

Inpatient data on childbirth deliveries were obtained from the University of North Carolina at Chapel Hill Cecil G. Sheps Center for Health Services Research. Hospitalization records were geocoded to each patient’s residential Zip Code Tabulation Area (ZCTA) based on the zip code recorded at admission, and the ZCTA served as the primary geographic unit of analysis for all spatial analysis and community-level variables. Delivery hospitalizations flagged using delivery-specific International Classification of Diseases (ICD) diagnosis codes (ICD-10: O80-O83) based on admission year. Pregnant persons characterized by multiple deliveries, a pregnancy with abortive outcomes, and those under the age of 18 years were excluded from our sample. The exclusion of individuals under age 18 is consistent with prior climate-sensitive SMM studies using the similar data sources (Harden et al., 2022; Sugg et al., 2023b, Ulrich et al., 2026), as adolescent pregnancies are shaped by distinct biological and social factors that warrant separate analytic consideration (Carr et al., 2022). Only individuals residing in an NC county, aged 18 or older, with a singleton birth were included. All analysis was conducted at the zip code tabulation area (ZCTA).

### Hospital Administrative Data

A widely disseminated algorithm, available through the Centers for Disease Control and Prevention (CDC), was used to identify 21 distinct indicators associated with severe maternal morbidity (SMM-21). These indicators were assessed while accounting for the duration of a patient’s hospital stay, occurrences of maternal mortality within the hospital setting, and the mode of delivery (e.g., repeat cesarean). Cases pertaining to ectopic or molar pregnancies, as well as hospitalizations for childbirth involving abortive procedures, were deliberately excluded from the analysis, as delineated in the CDC’s guidelines (Centers for Disease Control and Prevention, 2018). A secondary measure, SMM-20, which excludes the blood transfusion indicator due to concerns about the accuracy of transfusion volume documentation in hospital records (Snowden et al., 2021), was also constructed and used in sensitivity analyses.

### Racial and Income Segregation

The Index of Concentration at the Extremes (ICE) was used to measure contextual racial and income segregation at the ZCTA level, calculated using American Community Survey (ACS) 5-year estimates (Krieger et al., 2016). ICE scores range from − 1 to + 1 and quantify the degree to which a geographic area is polarized toward one demographic or socioeconomic extreme. Negative values indicate areas concentrated with low-income or non-White (predominantly Black or Hispanic) residents, while positive values indicate areas concentrated with high-income or White non-Hispanic residents; values near zero reflect mixed or unpolarized neighborhoods (Massey, 1996; Krieger et al., 2016). Two ICE measures were constructed: (1) ICE Race, which contrasts the proportion of White non-Hispanic residents against the proportion of Black and non-White Hispanic residents within each ZCTA; and (2) ICE Income, which contrasts households earning ≥$100,000 per year against households earning ≤$25,000 per year. Both measures were divided into tertiles: Q1 (predominantly non-White or low-income neighborhoods), Q2 (mixed neighborhoods), and Q3 (predominantly White or high-income neighborhoods), with Q3 serving as the reference category in all regression models. This tertile approach aligns with prior maternal health research in North Carolina using ICE (Sugg et al., 2025b; Ulrich et al., 2025). In disaster contexts, racial and economic segregation shape differential exposure and recovery, as communities in high-ICE-deprivation areas are disproportionately located in hazard-prone zones and face greater barriers to post-disaster healthcare access (Handwerger et al., 2021; Zanocco et al., 2022; Levy & Patz, 2015).

### Greenspace

Access to greenspace has been suggested as a factor contributing to improved birth outcomes (Woods et al., 2017; Runkle et al., 2022; Kihal-Talantikite et al., 2013) and child mental health during climate events (Sewell et al., 2024). Proposed potential mechanisms include stress reduction, improved air quality, and the promotion of physical activity and social cohesion (Runkle et al. 2022; Ryan et al. 2023a, b; Woods et al. 2017). In this study, the availability of greenspace was measured using the proportion of greenspace per person at the ZCTA level using cross-sectional data from PAD-US (Runkle et al., 2022).

### Rurality

Residence in rural areas has been examined as a risk factor for adverse maternal health (Ondusko et al., 2022, Sugg et al., 2025b) and maternal and perinatal mental health outcomes (Mollard et al., 2016). Rural and urban differences as a contributing factor to maternal health clustering were investigated using Rural-Urban Commuting Area (RUCA) codes at the ZCTA level, and were aggregated into three levels to classify each ZCTA as urban (codes < 4), suburban (codes 4 to 6), or rural (codes 7 to 10) as a measure of the rural-urban continuum (USDA ERS, 2020).

### Spatial Analysis

SaTScan has been widely applied in epidemiological research to detect spatial clusters of disease and health disparities (Kirby et al., 2017), including studies of maternal and child health outcomes (Tesema et al., 2020), veterinary and zoonotic disease surveillance (Arjkumpa et al., 2020), urban mental health and social disadvantage (Gruebner et al., 2015), and crisis-related behavioral health outcomes (Sugg et al., 2025a, Lucero et al., 2024). This study applied a Bernoulli model using Kulldorff’s spatial scan statistic (Kulldorff, 1997), which compares the case location distribution to the control location distribution and is independent of the underlying population distribution (Kulldorff, 2022; Sewell et al., 2024; Sugg et al., 2025a). Cases were defined as delivery hospitalizations complicated by SMM-21 during the post-Hurricane Florence period (09/14/2018–06/21/2019), corresponding to the 280-day gestation window of potential storm exposure. Controls were SMM-21 delivery hospitalizations from the corresponding pre-hurricane period (09/14/2017–06/21/2018) to account for seasonal birth patterns. Our rationale for this temporal case-control design was twofold: to detect spatial shifts in SMM burden coinciding with hurricane exposure, and to constrain the study window to pre-COVID-19 periods, as the pandemic onset in early 2020 would introduce substantial confounding. We acknowledge that this design identifies locations where the relative concentration of SMM shifted between periods rather than isolating hurricane-attributable effects. To ensure the robustness of the findings, multiple maximum window sizes were evaluated, ranging from 5% to 50% of the at-risk population in 5% increments (Sokale et al. 2023; Terefe et al. 2022; Sugg et al. 2023a, 2016b). A 15% window was selected because results persisted across adjacent specifications (10%, 15%, 20%), consistent with sensitivity approaches in related SaTScan studies (Lucero et al. 2024; Ryan et al. 2023a, b). The maximum spatial cluster window size defines the upper limit on the percentage of the total at-risk population that can fall within a single scanning window (Kulldorff, 1997). Primary and secondary clusters were identified from likelihood ratio tests using p-values (*p* < 0.05) and 999 Monte Carlo replications (Kulldorff 2006).

### Multivariable Logistic Regression

Spatial clusters identified in the exposed cohort using SatScan were merged with individual-level delivery hospitalizations for SMM and community-level factors at the ZCTA level to perform a multivariable logistic regression. Separate regression models were estimated for SMM-21 and SMM-20, each with individual (e.g., age, race) and community-level (e.g., ICE, green space) variables, as well as FEMA disaster-declared residence (e.g., indicated as YES/NO) for each individual maternal health outcome. A multivariable logistic regression with maternal residence in a high-risk cluster (0/1) as the dependent variable was used to investigate associations between patient-level and community risk factors and the SMM spatial clustering of each ZCTA. ICE Race, ICE Income, greenspace, and RUCA levels were grouped into tertiles. AIC-based stepwise selection was used to identify a parsimonious model. Variables were evaluated for multicollinearity using the variance inflation factor (VIF), and all independent variables with a VIF of less than two were included in the final model. All statistical analyses were performed using R statistical software and RStudio (version 4.2.3).

## Results

### Descriptive Statistics

Our sample comprised 2,894 childbirth hospitalizations for pregnant individuals in North Carolina. Demographic characteristics are summarized for all SMM delivery hospitalizations in the pre- and post-Florence periods [Table 1]. After the hurricane, more Black or African American (3.7% increase), and fewer White (1.2% decrease), Asian (0.1% decrease), and Other Race (1.2% decrease) individuals, experienced an SMM-related childbirth delivery event. There was no significant difference in the average age of SMM patients, with a mean age of 28 in both pre- and post-hurricane exposure groups. The total number of delivery hospitalizations complicated by SMM was 1,522 during the pre-hurricane Florence period and 1,372 during the post-hurricane Florence period.

**Table 1:** Sample characteristics by hurricane exposure period

Characteristic	Overall(*N* = 2,894)	Post-hurricane(*n* = 1,372)	Pre-hurricane(*n* = 1,522)	*p*	SMD
Age, years — mean (SD)	28.45 (6.34)	28.22 (6.34)	28.66 (6.34)	0.064	0.069
Age category, n (%)					
Teenage (≤ 19)	205 (7.1)	105 (7.7)	100 (6.6)		
Early 20 s (20–24)	670 (23.2)	318 (23.2)	352 (23.1)	0.506	0.077
Mid 20 s (25–29)	766 (26.5)	374 (27.3)	392 (25.8)		
Early 30 s (30–34)	705 (24.4)	332 (24.2)	373 (24.5)		
Late 30 s (35–39)	422 (14.6)	184 (13.4)	238 (15.6)		
Age ≥ 40	126 (4.4)	59 (4.3)	67 (4.4)		
Race/ethnicity, n (%)					
White	1,280 (44.2)	598 (43.6)	682 (44.8)	**0.013**	0.150
Black or African American	1,007 (34.8)	504 (36.7)	503 (33.0)		
Asian	80 (2.8)	37 (2.7)	43 (2.8)		
American Indian/Pacific Islander†	72 (2.5)	23 (1.7)	49 (3.2)		
Other race	349 (12.1)	157 (11.4)	192 (12.6)		
Declined or unavailable	106 (3.7)	53 (3.9)	53 (3.5)		
ICE income tertile, n (%)					
Q3: Predominantly high-income neighborhoods	949 (33.3)	443 (32.8)	506 (33.8)	0.756	0.028
Q2: Medium	949 (33.3)	459 (34.0)	490 (32.7)		
Q1: Predominantly low-income neighborhoods	950 (33.4)	449 (33.2)	501 (33.5)		
ICE race tertile, n (%)					
Q3: Predominantly white neighborhoods	949 (33.3)	447 (33.1)	502 (33.5)	0.447	0.048
Q2: Medium	950 (33.3)	439 (32.5)	511 (34.1)		
Q1: Predominantly non-white neighborhoods	950 (33.3)	466 (34.5)	484 (32.3)		
Greenspace tertile, n (%)					
Low	898 (33.3)	443 (34.7)	455 (32.1)	0.254	0.064
Medium	898 (33.3)	427 (33.4)	471 (33.3)		
High	898 (33.3)	408 (31.9)	490 (34.6)		
RUCA level, n (%)					
Urban	2,142 (75.2)	1,027 (76.0)	1,115 (74.5)	0.588	0.039
Suburban	485 (17.0)	220 (16.3)	265 (17.7)		
Rural	222 (7.8)	105 (7.8)	117 (7.8)		
FEMA designation, n (%)					
No	1,836 (65.2)	870 (65.0)	966 (65.3)	0.881	0.007
Yes	982 (34.8)	469 (35.0)	513 (34.7)		

Pre-hurricane: 09/14/2017–06/21/2018. Post-hurricane: 09/14/2018–06/21/2019. *N* = 2,894 total delivery hospitalizations with SMM

*SMD* standardized mean difference. p-values from chi-square tests (categorical) or t-test (continuous). Bold p-value indicates significant difference between groups (*p* < 0.05)

†American Indian/Pacific Islander combines American Indian or Alaska Native (*n* = 67) and Native Hawaiian or Pacific Islander (*n* = 5) due to small cell sizes; group-specific comparisons should be interpreted with caution

### Clustering Analysis for SMM

**Fig. 1:**
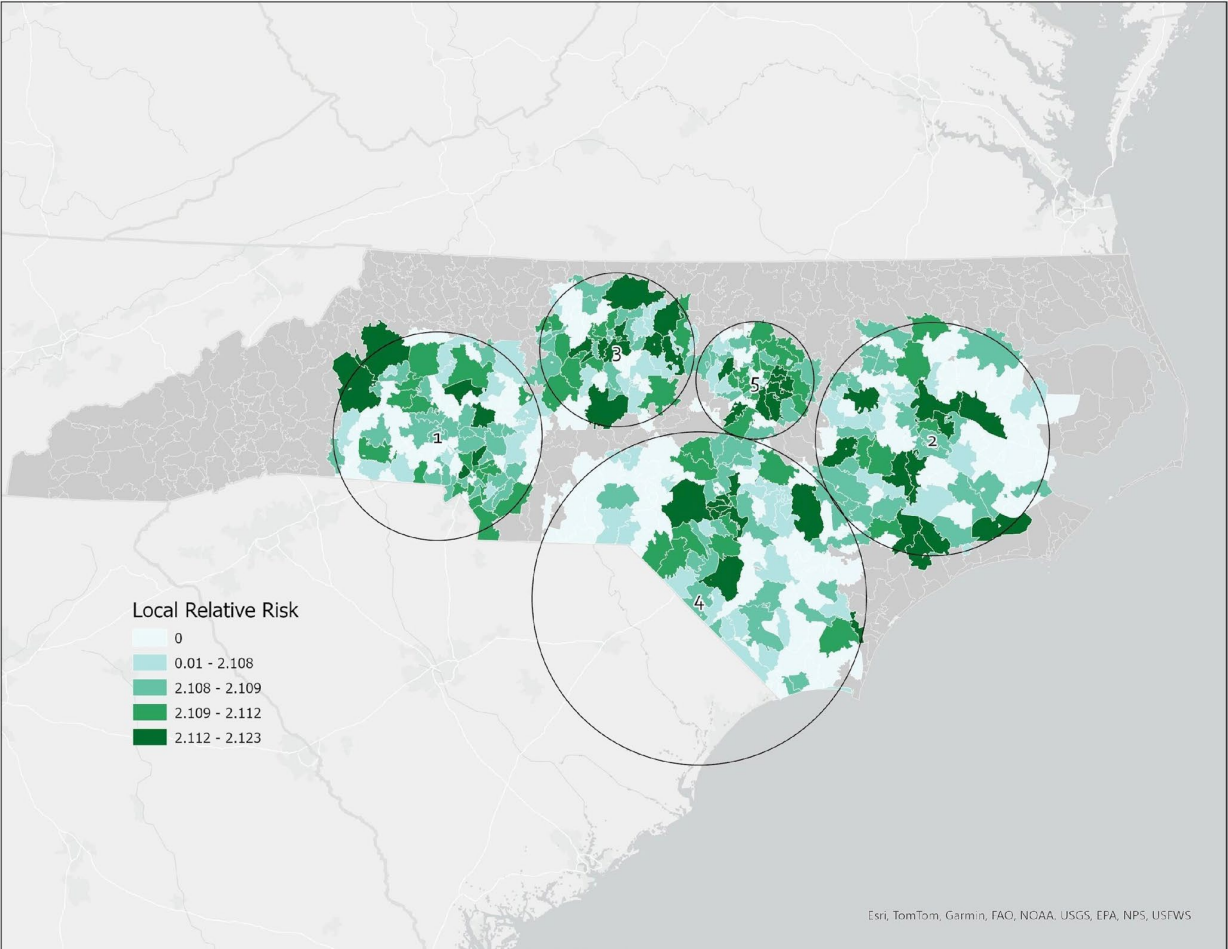
Bernoulli distribution of spatial cluster locations and cluster relative risk for severe maternal morbidity (SMM-21) identified in the post-hurricane Florence exposure period. Relative risk (RR) is defined by the ratio of the risk for an event in the exposure group to the risk for the non-exposure group. Five clusters were identified: Cluster 1, Charlotte metropolitan area (RR = 2.33); Cluster 2, Eastern North Carolina (RR = 2.32); Cluster 3, Greensboro/Triad area (RR = 2.32); Cluster 4, Lumberton/southeastern North Carolina (RR = 2.31); and Cluster 5, Raleigh metropolitan area (RR = 2.28). Cluster boundaries represent the circular scanning window used in SaTScan and do not correspond to administrative boundaries. Relative risk (RR) is defined as the ratio of SMM risk within the cluster to SMM risk outside the cluster. Non-cluster areas are not shown

The Bernoulli SaTScan analysis identified 5 significant clusters with increased SMM prevalence during the post-hurricane Florence period [Figure 1]. Clusters encompassed 471 Zip Code Tabulation Areas (ZCTAs). Overall, the relative risks for the 5 clusters ranged in (RR) from 2.28 to 2.33 (p-value < 0.05). The most likely cluster was identified in the Charlotte metropolitan area (RR = 2.33), followed by Eastern North Carolina (RR = 2.32), the Greensboro/Triad area (RR = 2.32), Lumberton/southeastern North Carolina (RR = 2.31), and the Raleigh metropolitan area (RR = 2.28). All clusters were statistically significant (*p* < 0.05). All clusters manifested during the same time period of post-hurricane exposure (9/14/2018- 6/21/2019). The population of clusters ranged from 185 to 226 individuals.

**Fig. 2:**
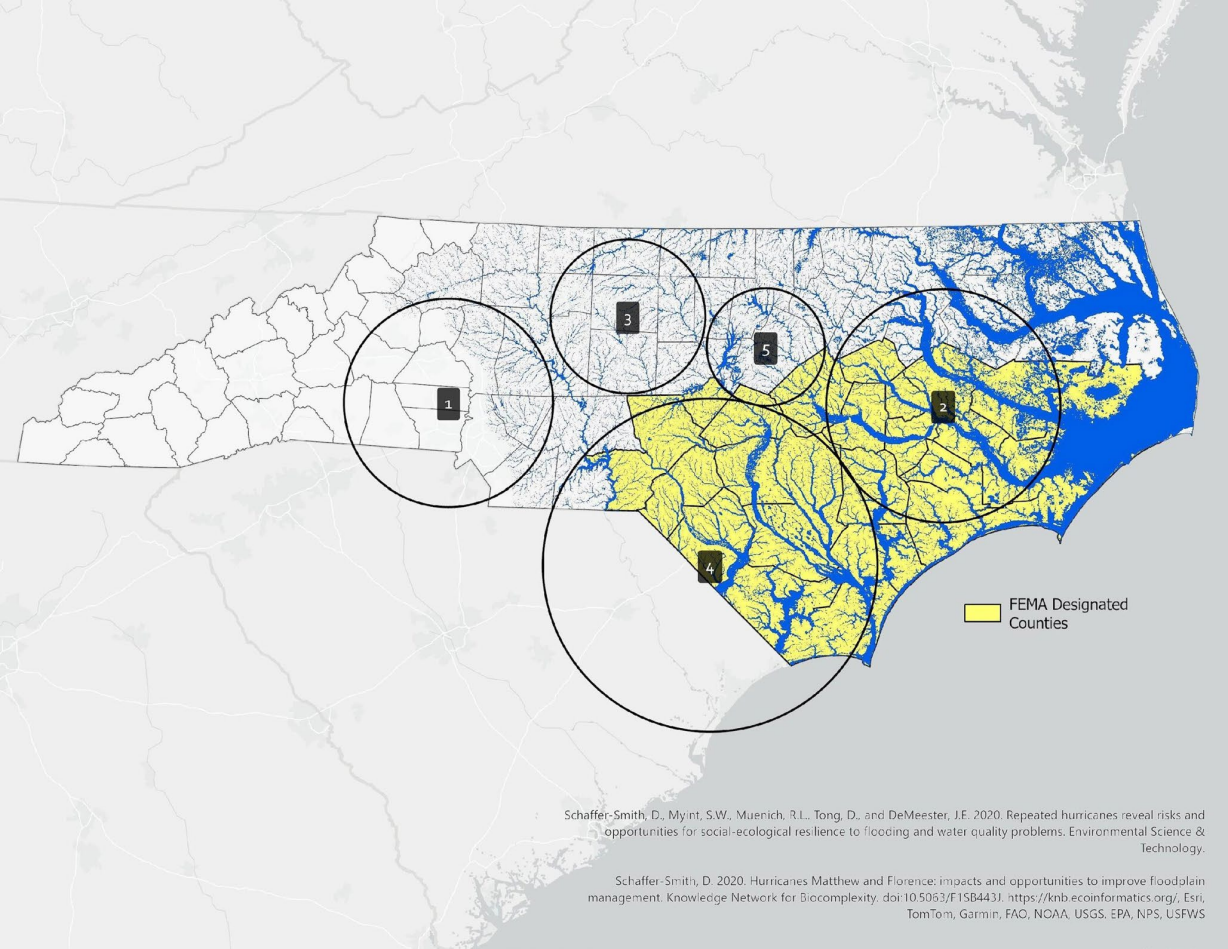
Hurricane Florence (2018) Flood Extent across the Piedmont and Coastal Plains of North Carolina (NC One Map, 2018) with Bernoulli distribution of spatial cluster locations for SMM-21 and FEMA-designated counties. Five clusters were identified: Cluster 1, Charlotte metropolitan area (RR = 2.33); Cluster 2, Eastern North Carolina (RR = 2.32); Cluster 3, Greensboro/Triad area (RR = 2.32); Cluster 4, Lumberton/southeastern North Carolina (RR = 2.31); and Cluster 5, Raleigh metropolitan area (RR = 2.28). Cluster boundaries represent the circular scanning window used in SaTScan and do not correspond to administrative boundaries. Relative risk (RR) is defined as the ratio of SMM risk within the cluster to SMM risk outside the cluster. Non-cluster areas are not shown

Under FEMA-DR-4393-NC, 28 NC counties were designated as disaster areas to receive Public Assistance (PA) and Individual Assistance (IA) after Hurricane Florence, and nearly 140,000 NC residents registered for assistance (FEMA, 2018; NOAA, 2018). In the Bernoulli distribution, all 28 counties listed as a FEMA disaster location were present in an SMM cluster [Figure 2].

### Multivariable Logistic Regression

Odds ratios (OR) and confidence intervals (CI) were found through logistic regression for individual-and-community-level factors associated with geographic high-risk spatial clusters for SMM among pregnant individuals post-Hurricane Florence [Table 2]. Individuals residing in a disaster-declared county were more likely to reside in an SMM cluster (OR = 2.02; 95% CI: 1.33–3.12) than those who were not, however results should be interpreted with caution due to different geographic scales (e.g., county vs. ZCTA). Racial segregation was the dominant predictor of SMM cluster residence: compared to predominantly White neighborhoods (ICE Race Q3), individuals residing in predominantly non-White neighborhoods (ICE Race Q1) had 9.31 times the odds of cluster residence (95% CI: 5.58–15.92), and those in mixed neighborhoods (ICE Race Q2) had 7.85 times the odds (95% CI: 5.01–12.60). Maternal residence in predominantly low-income ZCTAs (ICE Income Q1) was associated with significantly lower odds of SMM cluster residence compared to predominantly high-income ZCTAs (ICE Income Q3) (OR = 0.41; 95% CI: 0.25–0.65). Age ≥ 40 years was significantly associated with cluster residence (OR = 3.67; 95% CI: 1.34–12.27). RUCA grouping indicated urban residence (OR = 6.38; 95% CI: 3.34–12.31) was significantly more likely to be associated with SMM cluster residence than rural residence. Areas with high greenspace availability were significantly less likely to be associated with cluster residence compared to areas with low greenspace (OR = 0.37; 95% CI: 0.25–0.55).

**Table 2:** Full multivariable logistic regression models for SMM cluster residence, post-Hurricane Florence

Predictor	SMM-21 full model			SMM-20 full model		
	OR	95% CI	*p*	OR	95% CI	*p*
Age (Reference = Early 20 s)						
Teenage (≤ 19)	1.03	0.53–2.05	0.931	1.56	0.94–2.58	0.083
Mid 20 s (25–29)	1.24	0.78–1.97	0.360	1.38	0.98–1.96	0.066
Early 30 s (30–34)	0.94	0.59–1.48	0.785	1.40	0.98–1.99	0.064
Late 30 s (35–39)	1.18	0.69–2.07	0.548	1.36	0.90–2.05	0.139
Age ≥ 40	3.67*	**1.34–12.27**	**0.019**	1.56	0.83–2.96	0.169
Race (Reference = White)						
American Indian†	5.26	0.83–105.77	0.142	0.09*	**0.00–0.48**	**0.023**
Asian	5.29*	**1.50–26.93**	**0.021**	2.33*	**1.13–4.98**	**0.024**
Black or African American	1.43	0.93–2.19	0.101	1.42*	**1.06–1.90**	**0.019**
Declined or unavailable	1.55	0.73–3.41	0.260	1.14	0.60–2.15	0.678
NHPI‡	–	–	–	–	–	–
Other race	2.08*	**1.15–3.94**	**0.019**	1.86**	**1.24–2.80**	**0.003**
ICE race (Reference = Q3: Predominantly white neighborhoods)						
Q1: Predominantly non-white neighborhoods	9.31***	**5.58–15.92**	**< 0.001**	3.39***	**2.38–4.87**	**< 0.001**
Q2: Medium	7.85***	**5.01–12.60**	**< 0.001**	1.83***	**1.33–2.52**	**< 0.001**
ICE income (Reference = Q3: Predominately high-income neighborhoods)						
Q1: Predominately low-income neighborhoods	0.41***	**0.25–0.65**	**< 0.001**	0.24***	**0.16–0.34**	**< 0.001**
Q2: Medium	0.74	0.49–1.14	0.170	0.77	0.57–1.03	0.080
Greenspace (Reference = Low)						
High	0.37***	**0.25–0.55**	**< 0.001**	0.65**	**0.48–0.88**	**0.005**
Medium	0.71	0.45–1.11	0.135	0.71*	**0.53–0.96**	**0.026**
RUCA (Reference = Rural)						
Suburban	1.48	0.76–2.89	0.249	2.12*	**1.16–3.96**	**0.016**
Urban	6.38***	**3.34–12.31**	**< 0.001**	1.17	0.66–2.12	0.604
FEMA disaster designation (Reference = No)						
Yes	2.02**	**1.33–3.12**	**0.001**	0.84	0.64–1.11	0.218

Outcome = binary residence within post-hurricane SaTScan cluster boundary. SMM-21: 5 clusters; SMM-20: 3 clusters (15% spatial window). *n* = 1,265 complete cases for both models. FEMA designation was removed by stepwise selection in the SMM-20 model (OR = 0.84; *p* = 0.218) and is retained in Table 2 for comparability

Bold = statistically significant (*p* < 0.05). Gray shading = non-significant predictor retained in model. All covariates retained in both models; FEMA was the only predictor removed by AIC-based stepwise selection in SMM-20 and is included here for completeness (OR = 0.84, *p* = 0.218)

NHPI‡ suppressed due to near-perfect separation (*n* ≤ 5). †American Indian estimate unstable in SMM-21 due to small cell size (wide CI)

*p < 0.05 **p < 0.01 ***p < 0.001. VIF < 2.0 for all predictors in both models. AIC: SMM-21 = 978.22; SMM-20 = 1600.64

### Sensitivity Analysis

**Fig. 3:**
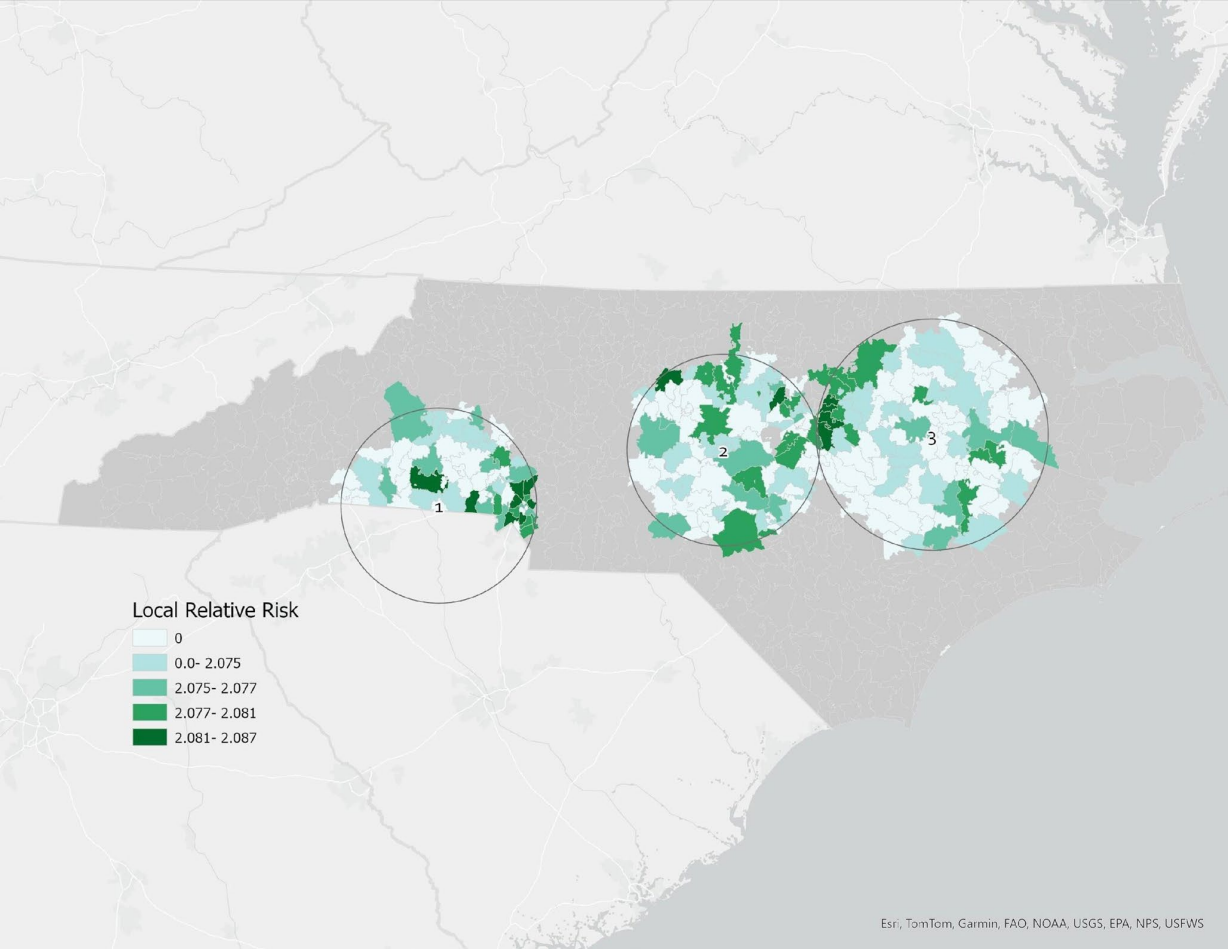
Bernoulli distribution of spatial cluster locations and cluster relative risk for severe maternal morbidity (SMM-20) identified in the post-hurricane Florence exposure period (9/1/2018- 6/21/2019). Relative risk (RR) is defined by the ratio of the risk for an event for the exposure group to the risk for the non-exposure group. Three clusters were identified: Cluster 1, western North Carolina/Charlotte area (RR = 2.32); Cluster 2, central Piedmont (RR = 2.29); and Cluster 3, eastern North Carolina (RR = 2.26). Cluster boundaries represent the circular scanning window used in SaTScan and do not correspond to administrative boundaries. Relative risk (RR) is defined as the ratio of SMM risk within the cluster to SMM risk outside the cluster. Non-cluster areas are not shown

A sensitivity analysis was performed to examine the robustness of the results. While some studies (Jiao et al., 2023) use SMM-21 as a primary severe maternal morbidity indicator, others (Snowden et al., 2021) omit the diagnosis of SMM linked to blood transfusions due to the inadequacy of hospital records in accurately documenting the volume of blood transferred. Therefore, additional analyses in both spatial and regression techniques were performed with severe maternal morbidity (SMM-20), which excludes blood transfusions. Using a SaTScan Bernoulli distribution at 15% window size, three statistically significant clusters were observed with increased SMM prevalence in the post-hurricane Florence period (September 2018- June 2019) [Figure 3]. Two clusters occurred in high-damage zones, and 18 of the 28 counties listed as FEMA disaster locations had cluster presence. Overall, the relative risks (RR) for the 3 clusters ranged from 2.26 to 2.32 (p-value < 0.05). The regression results from this sensitivity analysis are presented in Table 2.

As an additional sensitivity analysis, the multivariable logistic regression was repeated on the pre-hurricane cohort using post-hurricane high-SMM cluster boundaries (Supplemental Table S1). Consistent with their time-invariant nature, community-level covariates (FEMA designation, ICE Income, ICE Race, RUCA, greenspace) predicted cluster residence in both periods. Black race was significantly associated with cluster residence pre-hurricane (OR = 1.82, 95% CI: 1.27–2.63) but decreased in the post-hurricane (OR = 1.43, 95% CI: 0.93–2.19). Several community-level associations, RUCA urbanicity (pre: OR = 5.11; post: OR = 6.38), high greenspace (pre: OR = 0.48; post: OR = 0.37, both protective), and FEMA designation (pre: OR = 1.50; post: OR = 2.02), strengthened post-hurricane.

## Discussion

To our knowledge, this exploratory analysis is the first to examine the spatial clustering of adverse maternal health conditions, as measured by severe maternal morbidity following Hurricane Florence (2018). As we hypothesized, the results show a higher-than-expected prevalence of SMM in select locations across North Carolina following Hurricane Florence (2018), and that cluster residence was significantly more likely in FEMA disaster-declared counties. We found higher odds of SMM-21 cluster occurrence for racially segregated, predominantly non-White, and urban neighborhoods, with ICE Race emerging as the dominant structural predictor. Notably, while all 28 FEMA disaster-declared counties fell within an SMM-21 cluster, three of the five identified clusters, Charlotte, Raleigh, and Greensboro/Triad, were located outside the disaster zone, suggesting that significant maternal health burden extends beyond the hurricane’s direct footprint and may reflect pre-existing structural risk factors such as income segregation and urbanicity. These findings demonstrate the value of a statewide spatial approach that captures both disaster-related and underlying patterns of maternal risk.

Our study identified high-risk clustering of SMM-21 across North Carolina, including both disaster-impacted coastal areas and non-disaster urban regions. Under FEMA-DR-4393-NC, 28 NC counties were identified as disaster locations (FEMA 2018), and all 28 listed as FEMA disaster locations had SMM-21 cluster presence. Furthermore, FEMA designation was a significant factor in the regression analysis, suggesting that a pregnant person residing in a disaster-declared county were more than twice as likely to live in an SMM-21 cluster. However, the near-complete spatial overlap between FEMA-designated counties and ZCTA-level clusters limits the independent interpretive value of this finding, and FEMA designation is best understood as a contextual indicator of storm impact severity rather than an independent predictor of SMM risk. A similar spatial pattern of clusters in the southeastern coastal region of NC has been found to be related to high-risk clustering of maternal mental health disorders between 2016 and 2019 (Ulrich et al., 2023). Findings highlight that these locations may be characterized by maternal populations at higher risk for pregnancy complications both during and outside of climate-related disasters. Nonetheless, these results are consistent with the literature suggesting that climate-induced tropical cyclone events negatively affect pregnant individuals (Atwoli et al., 2021; IPCC, 2023; Silva-Suarez et al., 2021).

Literature suggests childbirth at a young (≤ 19 years old) or older pregnant persons associated with an increased risk of adverse maternal health and child outcomes (Kim et al., 2021, 2022; Aoyama, Pinto, and Ray, 2019; Carr et al., 2022). Consistent with the literature, our study found an increased risk of SMM cluster residence for pregnant individuals aged ≥ 40. This finding aligns with recent evidence from North Carolina demonstrating that higher maternal age (> 35) is associated with elevated SMM risk during extreme temperature events, suggesting that older maternal age may represent a consistent vulnerability across multiple climate-related and disaster contexts (Ulrich et al., 2026).

Our finding that urban areas were significantly more likely to be identified in high-risk clusters for SMM-21 is contrary to previous literature, which has documented rural residence as a risk factor for maternal health care due to limited access to health care, income disparities, fewer resources, transportation barriers, and underlying social determinants of health (MAHEC, 2022; Baxley, 2023). However, previous literature also notes that race and income may affect the relationship between rural-urban status and adverse maternal health outcomes at the national level; individuals in the most urban and most rural areas had higher odds of SMM (Kozhimannil et al. 2019a, 2019b; Luke et al. 2021). Our work emphasizes the need for further research investigating maternal health disparities along the rural-urban continuum for natural disasters like Hurricane Florence.

Racial segregation was the dominant predictor of SMM-21 cluster residence, with individuals in predominantly non-White neighborhoods at significantly elevated odds of cluster residence compared to predominantly White neighborhoods. Racial segregation has been broadly predictive of adverse health outcomes (Mehra et al., 2017; Williams and Lawrence, 2019), and Black individuals in the United States have consistently been found to be at higher risk of both SMM and SMM cluster residence (Hailu et al., 2022; Luke et al., 2021; Harden et al., 2022). This finding is consistent with prior NC-specific research demonstrating that ICE Race is a robust predictor of adverse maternal health clustering, including perinatal mood disorders, severe mental illness, and new-onset maternal mental disorders (Ulrich et al., 2023), as well as preterm birth, low birth weight, gestational diabetes, and pregnancy-induced hypertension (Ulrich et al., 2025), demonstrating the persistent role of structural racism in shaping maternal health geography across a range of adverse outcomes.

Notably, ICE Income showed a counterintuitive inverse association with SMM-21 cluster residence, with predominantly low-income neighborhoods having lower odds of cluster residence than high-income neighborhoods. This finding may reflect the spatial distribution of delivery hospitals, differential care-seeking patterns, or the concentration of hospital-based SMM detection in higher-income urban areas. Within North Carolina specifically, ICE Income has shown varying directional associations depending on the outcome examined, significantly predicting new-onset mental health conditions during pregnancy but not pre-existing conditions (Ulrich et al., 2023), and demonstrating stronger positive associations with birth outcomes such as preterm birth and low birth weight rather than maternal outcomes like gestational diabetes and pregnancy-induced hypertension (Ulrich et al., 2025). This variability supports the interpretation that income segregation and racial segregation operate through distinct pathways in shaping maternal health risk, and that their effects may be further modified by the post-disaster context of Hurricane Florence. Future research should consider a combined ICE Race and ICE Income analysis to better disentangle the structural factors driving SMM-21 clustering in disaster-affected populations.

Previous studies have used spatial analysis for assessing crime (Leitner and Helbich 2011), post-traumatic stress and depression (Gruebner et al. 2016a, 2016b), mental health wellness (Gruebner et al. 2016a, 2016b), and substance abuse (Moise and Ruiz 2016) following tropical cyclones. Our study is unique in applying a spatial approach to maternal health outcomes, which, to our knowledge, has not yet been done. Further research at local scales should consider clustering by vulnerable subgroups, trimester-specific analyses using linked vital statistics and hospital discharge data, additional mental health indicators, resource availability, and relocation due to exposure to tropical cyclones. Future studies should also analyze SMM as a proportion of all hospitalizations for delivery and incorporate continuous local relative risk rather than binary cluster membership to capture within-cluster heterogeneity.

### Strengths and Limitations

This study’s strengths include the use of validated ICD-10 maternal surveillance metrics and a subcounty-level spatial clustering approach that enables small-area analysis beyond the county-level resolution of prior work (Nagasoka et al., 2018).

Several limitations warrant consideration. SMM is a rare outcome, and small sample sizes may inflate relative risk estimates and limit generalizability; small cell sizes for specific racial groups (e.g., Native Hawaiian or Pacific Islander, American Indian) further restrict group-specific inference. Individual-level race associations should be interpreted cautiously, as compositional shifts in the delivery cohort between periods, such as changes in care-seeking following Florence, may explain observed differences (e.g., Black race: OR = 1.82, *p* = 0.001 pre-hurricane vs. OR = 1.43, *p* = 0.101 post-hurricane). Hospital administrative data lack individual-level income, marital status, and family structure, and North Carolina does not provide linked vital statistics records, precluding trimester-specific analyses or precise gestational age estimation at the time of exposure.

A key assumption of the Bernoulli temporal case-control design is that, absent the hurricane, the spatial distribution of SMM risk would be consistent across periods; accordingly, this design detects shifts in relative geographic concentration rather than isolating hurricane-attributable effects. The pre-hurricane sensitivity analysis (Supplemental Table S1) found community-level associations stable across periods by design, while post-hurricane amplification of RUCA urbanicity, greenspace, and FEMA associations is consistent with Florence intensifying pre-existing structural inequities. Additional methodological limitations include the use of binary cluster membership, which sacrifices the granularity of SaTScan’s continuous local relative risk (Sewell et al. 2024; Sugg et al. 2025a; Ryan et al. 2023a, 2023b); a geographic unit mismatch between county-level FEMA designations and ZCTA-level clusters; potential confounding from prior regional storms, including Hurricane Matthew (2016); and an inability to identify individuals displaced by Florence who may have faced reduced healthcare access (WHO, 2014).

## Conclusions

This study demonstrates the utility of spatial clustering methods for identifying geographic concentrations of severe maternal morbidity following a tropical cyclone. Findings suggest that both disaster-impacted and structurally disadvantaged communities experience elevated SMM clustering, with racial segregation, urbanicity, and age ≥ 40 emerging as strong predictors of high-risk cluster residence. These results can inform targeted post-disaster maternal health surveillance, resource allocation, and preparedness planning. As tropical cyclone frequency and intensity rise with climate change, spatially informed approaches will be essential for identifying and supporting high-burden maternal populations before, during, and after these events.

## Supplementary Information

Below is the link to the electronic supplementary material.

Supplementary file1 (DOCX 21 kb)

## Data Availability

Data were obtained from the University of North Carolina at Chapel Hill Cecil G. Sheps Center for Health Services Research through a data use agreement.

## Code Availability

R Studio version 4.3.0. SaTScan Statistical Software. ArcGIS Pro.
