# Supplementary material for: A Spatial Analytic Approach to Maternal Health Following Hurricane Florence (2018)
Source: Matern Child Health J. 2026 Apr 29;30(5):628–41. doi: 10.1007/s10995-026-04257-0 (PMC13190442; doi:10.1007/s10995-026-04257-0)
Supplement: Supplementary file 1 — Supplementary file1 (DOCX 21 kb) [file 10995_2026_4257_MOESM1_ESM.docx]

## **Appendix A. Supplemental Materials** (Attached)

**Supplemental Table S1.** Post- vs. Pre-Hurricane Full Logistic Regression Models — SMM-21. Outcome = binary residence within post-hurricane SMM-21 SaTScan cluster boundaries applied to both cohorts. Full models with all covariates retained.

| **Predictor** | **Post-Hurricane SMM-21(n=1,265; R²=0.308)** | | | **Pre-Hurricane SMM-21(n=1,398; R²=0.296)** | | |
| --- | --- | --- | --- | --- | --- | --- |
|  | **OR** | **95% CI** | **p** | **OR** | **95% CI** | **p** |
| **Age (Reference = Early 20s)** | | | | | | |
| Teenage (≤19) | 1.03 | 0.53–2.05 | 0.931 | 0.92 | 0.50–1.72 | 0.794 |
| Mid 20s (25–29) | 1.24 | 0.78–1.97 | 0.360 | 0.88 | 0.58–1.33 | 0.544 |
| Early 30s (30–34) | 0.94 | 0.59–1.48 | 0.785 | 1.00 | 0.65–1.52 | 0.991 |
| Age 35–39 | 1.18 | 0.69–2.07 | 0.548 | 0.77 | 0.48–1.23 | 0.267 |
| Age ≥40 | **3.67*** | **1.34–12.27** | **0.019** | 1.79 | 0.79–4.44 | 0.184 |
| **Race (Reference = White)** | | | | | | |
| American Indian† | 5.26 | 0.83–105.77 | 0.142 | 0.80 | 0.37–1.72 | 0.566 |
| Asian | **5.29*** | **1.50–26.93** | **0.021** | **12.09**** | **3.37–78.31** | **0.001** |
| Black or African American | 1.43 | 0.93–2.19 | 0.101 | **1.82**** | **1.27–2.63** | **0.001** |
| Declined or Unavailable | 1.55 | 0.73–3.41 | 0.260 | **0.49*** | **0.24–0.99** | **0.048** |
| NHPI‡ | — | — | — | — | — | — |
| Other Race | **2.08*** | **1.15–3.94** | **0.019** | **2.80***** | **1.69–4.82** | **<0.001** |
| **ICE Race (Reference = Q3: Predominantly White neighborhoods)** | | | | | | |
| Q1: Predominantly Non-White neighborhoods | **9.31***** | **5.58–15.92** | **<0.001** | **6.98***** | **4.56–10.85** | **<0.001** |
| Q2: Medium | **7.85***** | **5.01–12.60** | **<0.001** | **4.48***** | **3.11–6.52** | **<0.001** |
| **ICE Income (Reference = Q3: Most Privileged)** | | | | | | |
| Q1: Predominately Low-Income neighborhoods | **0.41***** | **0.25–0.65** | **<0.001** | **0.60*** | **0.39–0.92** | **0.018** |
| Q2: Medium | 0.74 | 0.49–1.14 | 0.170 | **0.69*** | **0.48–1.00** | **0.049** |
| **Greenspace (Reference = Low)** | | | | | | |
| High | **0.37***** | **0.25–0.55** | **<0.001** | **0.48***** | **0.33–0.68** | **<0.001** |
| Medium | 0.71 | 0.45–1.11 | 0.135 | 1.01 | 0.68–1.50 | 0.949 |
| **RUCA (Reference = Rural)** | | | | | | |
| Suburban | 1.48 | 0.76–2.89 | 0.249 | 0.89 | 0.50–1.58 | 0.684 |
| Urban | **6.38***** | **3.34–12.31** | **<0.001** | **5.11***** | **2.86–9.16** | **<0.001** |
| **FEMA Disaster Designation (Reference = No)** | | | | | | |
| Yes | **2.02**** | **1.33–3.12** | **0.001** | **1.50*** | **1.05–2.16** | **0.026** |

Bold = statistically significant (p<0.05). †American Indian estimate may be unstable due to small cell size. ‡NHPI suppressed: near-perfect separation (n≤5).

*p<0.05 **p<0.01 ***p<0.001.

Pre-hurricane cohort: 09/14/2017–06/21/2018 (n=1,398 complete cases). Post-hurricane cohort: 09/14/2018–06/21/2019 (n=1,265 complete cases). Community-level covariates (ICE Income, ICE Race, Greenspace, RUCA, FEMA) are time-invariant ZCTA-level measures; pre/post stability by construction.
